# 2-(1,3-Dioxoisoindolin-2-yl)ethyl 4-methyl­benzene­sulfonate

**DOI:** 10.1107/S1600536808037951

**Published:** 2008-11-20

**Authors:** Mark Daniel Bartholomä, Wayne Ouellette, Jon Zubieta

**Affiliations:** a Department of Chemistry, Syracuse University, Syracuse, New York 13244, USA

## Abstract

In the title mol­ecule, C_17_H_15_NO_5_S, the dihedral angle between the essentially planar atoms of the tosyl moiety (the S atom and the seven tolyl C atoms) and the phthalimide moiety is 6.089 (3)°. The mol­ecule is folded about the ethyl­ene bridge, adopting a staggered conformation such that the benzene ring of the tosyl group and the five-membered ring of the phthalimide moiety have a face-to-face orientation with a centroid-to-centroid separation of 3.7454 (12) Å. The crystal structure is stabilized by weak inter­molecular π–π inter­actions between symmetry-related five-membered rings of the phthalimide groups, with a centroid-to-centroid distance of 3.3867 (11) Å. The compound is used for the attachment of a suitable chelate functionality for radiolabeling purposes.

## Related literature

For general background, see: Eriksson *et al.* (2002 ▶); Arner & Eriksson (1995 ▶); Bello (1974 ▶); Wei *et al.* (2005 ▶); Welin *et al.* (2004 ▶). For reference bond distances, see: Allen *et al.* (1987 ▶).
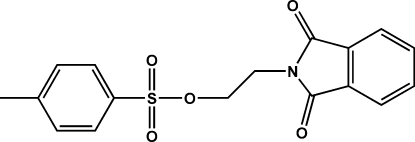


## Experimental

### 

#### Crystal data

C_17_H_15_NO_5_S
*M* *_r_* = 345.36
Monoclinic, 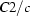

*a* = 13.6817 (13) Å
*b* = 12.5642 (12) Å
*c* = 19.3194 (19) Å
β = 107.121 (2)°
*V* = 3173.8 (5) Å^3^
*Z* = 8
Mo *K*α radiation
μ = 0.23 mm^−1^
*T* = 90 (2) K
0.40 × 0.35 × 0.30 mm

#### Data collection

Bruker APEX CCD area-detector diffractometer
Absorption correction: multi-scan (*SADABS*; Brucker, 2007 ▶) *T*_min_ = 0.913, *T*_max_ = 0.934
16184 measured reflections
3865 independent reflections
3773 reflections with *I* > 2σ(*I*)
*R*_int_ = 0.020

#### Refinement

*R*[*F* ^2^ > 2σ(*F* ^2^)] = 0.052
*wR*(*F* ^2^) = 0.122
*S* = 1.25
3865 reflections
218 parameters
H-atom parameters constrained
Δρ_max_ = 0.48 e Å^−3^
Δρ_min_ = −0.41 e Å^−3^

### 

Data collection: *SMART* (Bruker, 2007 ▶); cell refinement: *SAINT* (Bruker, 2007 ▶); data reduction: *SAINT*; program(s) used to solve structure: *SHELXS97* (Sheldrick, 2008 ▶); program(s) used to refine structure: *SHELXL97* (Sheldrick, 2008 ▶); molecular graphics: *CrystalMaker* (Palmer, 2006 ▶); software used to prepare material for publication: *SHELXTL* (Sheldrick, 2008 ▶).

## Supplementary Material

Crystal structure: contains datablocks I, global. DOI: 10.1107/S1600536808037951/lh2734sup1.cif

Structure factors: contains datablocks I. DOI: 10.1107/S1600536808037951/lh2734Isup2.hkl

Additional supplementary materials:  crystallographic information; 3D view; checkCIF report

## Figures and Tables

**Fig. 1.**
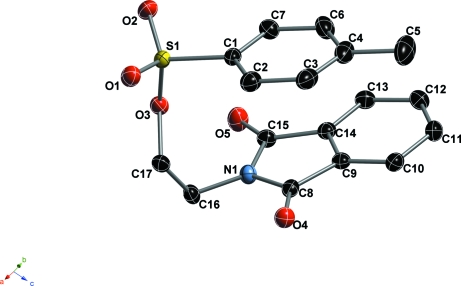
Perspective view of (I), with the atom numbering scheme and thermal ellipsoids drawn at 50% probability level.

**Fig. 2.**
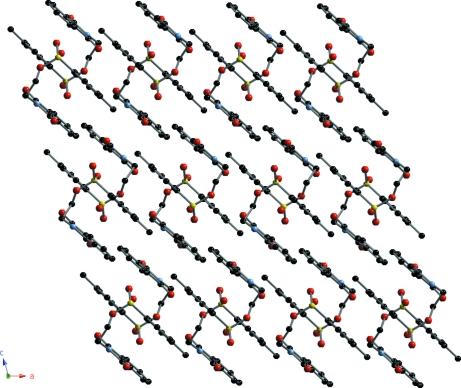
The crystal packing of the title compound viewed parallel to the ac plane.

## supplementary crystallographic information

### Comment

Nucleosides and nucleoside derivatives have become a target of interest as potential inhibitors and probes for tumor cell proliferation. One major targeted enzyme is the human cytosolic thymidine kinase (hTK-1), an enzyme of the pyrimidine salvage pathway which catalyzes the phosphorylation of nucleosides to their corresponding 5'-monophosphates (Welin *et al.*, 2004). The hTK-1 activity is closely related to DNA synthesis and the corresponding monophosphates are important precursors for DNA incorporation. Interestingly, hTK-1 shows a dramatically increased activity in proliferating cells compared to quiescent cells which makes it an attractive target for radiolabeling applications (Bello, 1974). Nucleosides are taken up by proliferating cells through facilitated diffusion and get converted to their corresponding monophosphates by hTK-1. The cellular efflux of the corresponding monophosphate is hindered due to the negatively charged phosphate residue leading to a intracellular trapping of the corresponding nucleoside (Arner & Eriksson, 1995). Thus, a radiolabeled nucleoside analog could be used as probe for tumor cell proliferation since the trapping results in an accumulation in tissue with elevated hTK-1 activity. Much effort has been put in the development of radiolabeled nucleoside analogs but the narrow substrate specifity of hTK-1 remains hereby a problem which still has to be solved (Eriksson *et al.*, 2002). The literature on the interaction of thymidine derivatives with hTK-1 is not totally unambiguous about the effects of various substitutions. Major modifications of thymidine or uridine, respectively, can result in inactivity. On the other hand, several derivatives modified at the ribose and the base site are reported which retain their activity. Therefore, we built up a library of several thymidine and uridine analogs modified at different positions of the sugar and base moiety to investigate the effects of various substitutions. 2-(1,3-dioxoisoindolin-2-yl)ethyl 4-methylbenzenesulfonate (Tosylethylphthalimide) is part of a series of tosylalkylphthalimide derivatives recently synthesized in our group. The series was prepared to expand the use of our SAAC concept (single amino acid chelate) on nucleosides for radioimaging and radiotherapeutic purposes (Bartholomä *et al.*, unpublished results). The tosylalkylphthalimide derivatives are precursors for the attachment of a SAAC chelate at the N-3 and C-5 position of the base moiety of thymidine. The SAAC chelate allows thereby the radiolabeling of thymidine and uridine derivatives by the coordination of the [*M*(CO)_3_]^+^ core (*M* = ^186/188^Re, ^99^*^m^*Tc) (Wei *et al.*, 2005). ^99^*^m^*Tc with its ideal decay properties, low cost and good availability can be used for imaging purposes while the corresponding rhenium complexes would be the therapeutic counterparts.

The title molecule shows a folded structure where the phthalimide residue and the tosyl moiety have a face-to-face orientation (see Fig. 1). Thereby, inter- as well as intramolecular aromatic interactions are observed. The intramolecular interactions are illustrated by the centroid-to-centroid distance between the five-membered ring of the phthalimide moiety and the benzene ring of the tosyl residue with Cg1···Cg2 = 3.7454 (3) Å, where Cg1 is the centroid of the ring atoms N1/C8/C9/C14/C15 and Cg2 is the centroid of the ring atoms C1–C7. Weak intermolecular interactions occur between two five-membered rings of the phthalimide moiety with a Cg1···Cg1^i^ distance of 3.3867 (3) Å [symmetry code: (i) -x, y, 3/2 - z]. The ethylene bridge adopts a low-energy staggered conformation with the torsion angle N1—C16—C17—O3 = 61.829 (5)°. Obviously, this arrangement allows a more dense crystal packing as the fully planar conformation (see Fig. 2). The phthalimide (N1/O4/O5/C8–C16) and the tosyl (S1/C1–C7) moiety are essentially planar and have an approximately parallel orientation with respect to each other giving a dihedral angle of 6.089 (3)°. All bond lengths fall in the expected ranges (Allen *et al.*,  1987).

### Experimental

8.00 g (41.84 mmol) *N*-(2-Hydroxyethyl)phthalimide were dissolved in 80 ml anhydrous pyridine under an inert atmosphere followed by a dropwise addition of 11.97 g (62.76 mmol, 1.5 equiv.) *p*-Toluenesulfonyl chloride in 80 ml anhydrous pyridine. After the addition was completed, the reaction mixture was stirred for additional 16 h. The reaction was quenched by the addition of ice. The crude reaction mixture was poured into an ice/water mixture resulting in a white precipitate which was extracted with 3 × 80 ml chloroform. The combined organic layers were washed with saturated sodium bicarbonate solution (150 ml) and twice with water, dried over anhydrous MgSO_4_, and finally evaporated to dryness. The product was obtained in good yields as a colourless amorphous powder (12.99 g, 90%). Single crystals suitable for X-ray diffraction were obtained by dissolving the product in an ethylacetate/methanol mixture 20:1 and storing the solution at 273 K for several days. ^1^H NMR (d6-DMSO): δ = 2.19 (s, 3 H), 3.80 (t, J = 4.91 Hz, 2 H), 4.28 (t, J = 4.95 Hz, 2 H), 7.15 (d, J = 7.98 Hz, 2 H), 7.55 (d, J = 8.25 Hz, 2 H), 7.55–7.83 (m, 4 H) p.p.m.. IR: ν = 3466, 3063, 2970, 2942, 1773, 1756, 1711, 1614, 1594, 1463, 1428, 1392, 1355, 1320, 1190, 176, 1119, 1093, 1041, 992, 913, 859, 811, 796, 768, 722, 704, 693, 668, 578, 552, 526, 493 cm^-1^.

### Refinement

H atoms were placed in calculated positions with C—H = 0.95–0.99 Å and included in the riding-model approximation with U_iso_(H) = 1.2U_eq_(C) or 1.5U_eq_(C) for methyl H atoms.

### Crystal data

**Table:**

C_17_H_15_NO_5_S	*F*_000_ = 1440
*M**_r_* = 345.36	*D*_x_ = 1.446 Mg m^−^^3^
Monoclinic, *C*2/*c*	Mo *K*α radiation λ = 0.71073 Å
Hall symbol: -C2yc	Cell parameters from 9534 reflections
*a* = 13.6817 (13) Å	θ = 2.2–28.3º
*b* = 12.5642 (12) Å	µ = 0.23 mm^−^^1^
*c* = 19.3194 (19) Å	*T* = 90 (2) K
β = 107.121 (2)º	Block, colourless
*V* = 3173.8 (5) Å^3^	0.40 × 0.35 × 0.30 mm
*Z* = 8	

### Data collection

**Table:**

Bruker APEX CCD area-detector diffractometer	3865 independent reflections
Monochromator: graphite	3773 reflections with *I* > 2σ(*I*)
Detector resolution: 512 pixels mm^-1^	*R*_int_ = 0.020
*T* = 90(2) K	θ_max_ = 28.1º
φ and ω scans	θ_min_ = 2.2º
Absorption correction: multi-scan(SADABS in SHELXL97; Sheldrick, 2008)	*h* = −18→18
*T*_min_ = 0.913, *T*_max_ = 0.934	*k* = −16→16
16184 measured reflections	*l* = −25→25

### Refinement

**Table:**

Refinement on *F*^2^	Secondary atom site location: difference Fourier map
Least-squares matrix: full	Hydrogen site location: inferred from neighbouring sites
*R*[*F*^2^ > 2σ(*F*^2^)] = 0.052	H-atom parameters constrained
*wR*(*F*^2^) = 0.122	*w* = 1/[σ^2^(*F*_o_^2^) + (0.0413*P*)^2^ + 6.2623*P*] where *P* = (*F*_o_^2^ + 2*F*_c_^2^)/3
*S* = 1.25	(Δ/σ)_max_ = 0.001
3865 reflections	Δρ_max_ = 0.48 e Å^−^^3^
218 parameters	Δρ_min_ = −0.41 e Å^−^^3^
Primary atom site location: structure-invariant direct methods	Extinction correction: none

### Special details

**Table:**

Geometry. All e.s.d.'s (except the e.s.d. in the dihedral angle between two l.s. planes) are estimated using the full covariance matrix. The cell e.s.d.'s are taken into account individually in the estimation of e.s.d.'s in distances, angles and torsion angles; correlations between e.s.d.'s in cell parameters are only used when they are defined by crystal symmetry. An approximate (isotropic) treatment of cell e.s.d.'s is used for estimating e.s.d.'s involving l.s. planes.
Refinement. Refinement of *F*^2^ against ALL reflections. The weighted *R*-factor *wR* and goodness of fit *S* are based on *F*^2^, conventional *R*-factors *R* are based on *F*, with *F* set to zero for negative *F*^2^. The threshold expression of *F*^2^ > σ(*F*^2^) is used only for calculating *R*-factors(gt) *etc*. and is not relevant to the choice of reflections for refinement. *R*-factors based on *F*^2^ are statistically about twice as large as those based on *F*, and *R*- factors based on ALL data will be even larger.

### Fractional atomic coordinates and isotropic or equivalent isotropic displacement parameters (Å^2^)

**Table:**

	*x*	*y*	*z*	*U*_iso_*/*U*_eq_	
S1	0.24768 (3)	−0.05689 (4)	1.04905 (2)	0.01844 (13)	
O1	0.27012 (11)	−0.16681 (11)	1.06516 (7)	0.0245 (3)	
O2	0.26255 (12)	0.01842 (12)	1.10672 (7)	0.0269 (3)	
O3	0.13136 (10)	−0.04377 (10)	1.00548 (7)	0.0195 (3)	
O4	0.12124 (10)	−0.09920 (11)	0.78928 (7)	0.0214 (3)	
O5	0.01573 (11)	0.16581 (11)	0.91348 (8)	0.0270 (3)	
N1	0.05468 (11)	0.01454 (12)	0.85773 (8)	0.0179 (3)	
C1	0.31279 (13)	−0.01266 (16)	0.98842 (10)	0.0193 (4)	
C2	0.35460 (15)	−0.08611 (17)	0.95184 (11)	0.0243 (4)	
H2	0.3486	−0.1603	0.9592	0.029*	
C3	0.40571 (15)	−0.04904 (19)	0.90397 (11)	0.0287 (4)	
H3	0.4350	−0.0988	0.8788	0.034*	
C4	0.41464 (15)	0.0590 (2)	0.89231 (11)	0.0294 (5)	
C5	0.46884 (19)	0.0980 (2)	0.83979 (13)	0.0425 (6)	
H5A	0.5003	0.0376	0.8223	0.064*	
H5B	0.5220	0.1491	0.8641	0.064*	
H5C	0.4196	0.1328	0.7987	0.064*	
C6	0.37176 (16)	0.13104 (18)	0.93012 (12)	0.0286 (4)	
H6	0.3773	0.2052	0.9226	0.034*	
C7	0.32129 (15)	0.09635 (16)	0.97844 (11)	0.0236 (4)	
H7	0.2930	0.1460	1.0043	0.028*	
C8	0.10819 (13)	−0.01011 (15)	0.80845 (9)	0.0169 (3)	
C9	0.14229 (13)	0.09383 (15)	0.78653 (9)	0.0180 (3)	
C10	0.19546 (15)	0.11612 (16)	0.73773 (10)	0.0218 (4)	
H10	0.2158	0.0612	0.7111	0.026*	
C11	0.21815 (16)	0.22272 (17)	0.72909 (11)	0.0270 (4)	
H11	0.2547	0.2410	0.6959	0.032*	
C12	0.18807 (17)	0.30273 (17)	0.76836 (11)	0.0276 (4)	
H12	0.2055	0.3745	0.7620	0.033*	
C13	0.13283 (15)	0.27955 (16)	0.81683 (10)	0.0232 (4)	
H13	0.1115	0.3341	0.8432	0.028*	
C14	0.11061 (13)	0.17402 (15)	0.82475 (9)	0.0182 (3)	
C15	0.05463 (14)	0.12411 (14)	0.87179 (10)	0.0186 (4)	
C16	0.00646 (14)	−0.06579 (15)	0.89112 (10)	0.0191 (4)	
H16A	−0.0302	−0.1175	0.8538	0.023*	
H16B	−0.0443	−0.0312	0.9112	0.023*	
C17	0.08415 (14)	−0.12402 (14)	0.95089 (10)	0.0191 (4)	
H17A	0.0505	−0.1797	0.9720	0.023*	
H17B	0.1364	−0.1581	0.9320	0.023*	

### Atomic displacement parameters (Å^2^)

**Table:**

	*U*^11^	*U*^22^	*U*^33^	*U*^12^	*U*^13^	*U*^23^
S1	0.0222 (2)	0.0191 (2)	0.0151 (2)	−0.00308 (16)	0.00727 (16)	−0.00143 (16)
O1	0.0290 (7)	0.0227 (7)	0.0213 (7)	−0.0010 (6)	0.0067 (5)	0.0018 (5)
O2	0.0362 (8)	0.0282 (7)	0.0185 (6)	−0.0083 (6)	0.0113 (6)	−0.0065 (6)
O3	0.0209 (6)	0.0188 (6)	0.0209 (6)	−0.0023 (5)	0.0095 (5)	−0.0037 (5)
O4	0.0227 (7)	0.0198 (6)	0.0233 (6)	0.0022 (5)	0.0094 (5)	−0.0020 (5)
O5	0.0325 (8)	0.0237 (7)	0.0304 (7)	0.0001 (6)	0.0180 (6)	−0.0042 (6)
N1	0.0182 (7)	0.0181 (7)	0.0191 (7)	0.0008 (6)	0.0083 (6)	0.0005 (6)
C1	0.0169 (8)	0.0260 (9)	0.0151 (8)	−0.0037 (7)	0.0048 (6)	0.0001 (7)
C2	0.0222 (9)	0.0288 (10)	0.0223 (9)	−0.0010 (8)	0.0071 (7)	−0.0032 (8)
C3	0.0201 (9)	0.0450 (13)	0.0220 (9)	0.0003 (8)	0.0079 (7)	−0.0044 (9)
C4	0.0169 (9)	0.0493 (13)	0.0222 (9)	−0.0054 (8)	0.0061 (7)	0.0040 (9)
C5	0.0297 (11)	0.0686 (18)	0.0340 (12)	−0.0074 (11)	0.0167 (10)	0.0104 (12)
C6	0.0242 (10)	0.0316 (11)	0.0303 (10)	−0.0067 (8)	0.0085 (8)	0.0063 (8)
C7	0.0216 (9)	0.0248 (10)	0.0244 (9)	−0.0022 (7)	0.0069 (7)	−0.0003 (7)
C8	0.0137 (7)	0.0219 (9)	0.0150 (8)	0.0014 (6)	0.0042 (6)	0.0013 (6)
C9	0.0158 (8)	0.0202 (8)	0.0165 (8)	−0.0012 (6)	0.0026 (6)	−0.0005 (7)
C10	0.0225 (9)	0.0267 (10)	0.0171 (8)	−0.0034 (7)	0.0071 (7)	−0.0018 (7)
C11	0.0300 (10)	0.0321 (11)	0.0207 (9)	−0.0095 (8)	0.0105 (8)	−0.0003 (8)
C12	0.0351 (11)	0.0227 (9)	0.0248 (9)	−0.0103 (8)	0.0084 (8)	−0.0005 (8)
C13	0.0269 (10)	0.0211 (9)	0.0212 (9)	−0.0037 (7)	0.0066 (7)	−0.0033 (7)
C14	0.0167 (8)	0.0212 (9)	0.0163 (8)	−0.0017 (7)	0.0045 (6)	−0.0019 (7)
C15	0.0181 (8)	0.0178 (8)	0.0197 (8)	0.0001 (6)	0.0053 (7)	−0.0015 (7)
C16	0.0182 (8)	0.0196 (8)	0.0216 (9)	−0.0026 (7)	0.0092 (7)	0.0002 (7)
C17	0.0219 (8)	0.0169 (8)	0.0190 (8)	−0.0040 (7)	0.0068 (7)	−0.0025 (7)

### Geometric parameters (Å, °)

**Table:**

S1—O1	1.4290 (15)	C6—C7	1.385 (3)
S1—O2	1.4299 (14)	C6—H6	0.9500
S1—O3	1.5747 (14)	C7—H7	0.9500
S1—C1	1.7574 (18)	C8—C9	1.489 (3)
O3—C17	1.465 (2)	C9—C10	1.379 (3)
O4—C8	1.209 (2)	C9—C14	1.392 (3)
O5—C15	1.208 (2)	C10—C11	1.396 (3)
N1—C8	1.396 (2)	C10—H10	0.9500
N1—C15	1.403 (2)	C11—C12	1.393 (3)
N1—C16	1.456 (2)	C11—H11	0.9500
C1—C2	1.384 (3)	C12—C13	1.396 (3)
C1—C7	1.393 (3)	C12—H12	0.9500
C2—C3	1.394 (3)	C13—C14	1.379 (3)
C2—H2	0.9500	C13—H13	0.9500
C3—C4	1.387 (3)	C14—C15	1.488 (2)
C3—H3	0.9500	C16—C17	1.509 (3)
C4—C6	1.396 (3)	C16—H16A	0.9900
C4—C5	1.504 (3)	C16—H16B	0.9900
C5—H5A	0.9800	C17—H17A	0.9900
C5—H5B	0.9800	C17—H17B	0.9900
C5—H5C	0.9800		
			
O1—S1—O2	119.84 (9)	O4—C8—C9	129.65 (17)
O1—S1—O3	109.64 (8)	N1—C8—C9	105.69 (15)
O2—S1—O3	103.69 (8)	C10—C9—C14	121.68 (18)
O1—S1—C1	109.41 (9)	C10—C9—C8	130.16 (17)
O2—S1—C1	109.05 (9)	C14—C9—C8	108.15 (16)
O3—S1—C1	103.97 (8)	C9—C10—C11	117.22 (18)
C17—O3—S1	118.36 (11)	C9—C10—H10	121.4
C8—N1—C15	112.32 (15)	C11—C10—H10	121.4
C8—N1—C16	123.05 (15)	C12—C11—C10	121.04 (18)
C15—N1—C16	124.62 (15)	C12—C11—H11	119.5
C2—C1—C7	121.40 (18)	C10—C11—H11	119.5
C2—C1—S1	119.74 (15)	C11—C12—C13	121.33 (19)
C7—C1—S1	118.85 (15)	C11—C12—H12	119.3
C1—C2—C3	118.6 (2)	C13—C12—H12	119.3
C1—C2—H2	120.7	C14—C13—C12	117.10 (18)
C3—C2—H2	120.7	C14—C13—H13	121.5
C4—C3—C2	121.3 (2)	C12—C13—H13	121.5
C4—C3—H3	119.3	C13—C14—C9	121.61 (17)
C2—C3—H3	119.3	C13—C14—C15	130.00 (17)
C3—C4—C6	118.61 (19)	C9—C14—C15	108.39 (16)
C3—C4—C5	120.9 (2)	O5—C15—N1	125.45 (17)
C6—C4—C5	120.5 (2)	O5—C15—C14	129.15 (17)
C4—C5—H5A	109.5	N1—C15—C14	105.40 (15)
C4—C5—H5B	109.5	N1—C16—C17	111.48 (15)
H5A—C5—H5B	109.5	N1—C16—H16A	109.3
C4—C5—H5C	109.5	C17—C16—H16A	109.3
H5A—C5—H5C	109.5	N1—C16—H16B	109.3
H5B—C5—H5C	109.5	C17—C16—H16B	109.3
C7—C6—C4	121.2 (2)	H16A—C16—H16B	108.0
C7—C6—H6	119.4	O3—C17—C16	106.23 (14)
C4—C6—H6	119.4	O3—C17—H17A	110.5
C6—C7—C1	118.77 (19)	C16—C17—H17A	110.5
C6—C7—H7	120.6	O3—C17—H17B	110.5
C1—C7—H7	120.6	C16—C17—H17B	110.5
O4—C8—N1	124.67 (17)	H17A—C17—H17B	108.7
			
O1—S1—O3—C17	−38.67 (14)	O4—C8—C9—C14	−178.67 (18)
O2—S1—O3—C17	−167.78 (12)	N1—C8—C9—C14	1.69 (19)
C1—S1—O3—C17	78.22 (14)	C14—C9—C10—C11	1.2 (3)
O1—S1—C1—C2	14.61 (18)	C8—C9—C10—C11	−179.09 (18)
O2—S1—C1—C2	147.43 (15)	C9—C10—C11—C12	0.0 (3)
O3—S1—C1—C2	−102.45 (16)	C10—C11—C12—C13	−1.0 (3)
O1—S1—C1—C7	−165.23 (15)	C11—C12—C13—C14	0.8 (3)
O2—S1—C1—C7	−32.41 (18)	C12—C13—C14—C9	0.4 (3)
O3—S1—C1—C7	77.71 (16)	C12—C13—C14—C15	179.45 (18)
C7—C1—C2—C3	−0.3 (3)	C10—C9—C14—C13	−1.4 (3)
S1—C1—C2—C3	179.90 (15)	C8—C9—C14—C13	178.79 (17)
C1—C2—C3—C4	−0.4 (3)	C10—C9—C14—C15	179.32 (16)
C2—C3—C4—C6	0.5 (3)	C8—C9—C14—C15	−0.45 (19)
C2—C3—C4—C5	−179.22 (19)	C8—N1—C15—O5	−178.31 (18)
C3—C4—C6—C7	0.1 (3)	C16—N1—C15—O5	0.7 (3)
C5—C4—C6—C7	179.75 (19)	C8—N1—C15—C14	2.1 (2)
C4—C6—C7—C1	−0.7 (3)	C16—N1—C15—C14	−178.90 (16)
C2—C1—C7—C6	0.8 (3)	C13—C14—C15—O5	0.3 (3)
S1—C1—C7—C6	−179.38 (15)	C9—C14—C15—O5	179.49 (19)
C15—N1—C8—O4	177.95 (17)	C13—C14—C15—N1	179.90 (19)
C16—N1—C8—O4	−1.1 (3)	C9—C14—C15—N1	−0.95 (19)
C15—N1—C8—C9	−2.38 (19)	C8—N1—C16—C17	76.8 (2)
C16—N1—C8—C9	178.62 (15)	C15—N1—C16—C17	−102.06 (19)
O4—C8—C9—C10	1.6 (3)	S1—O3—C17—C16	−146.02 (12)
N1—C8—C9—C10	−178.05 (18)	N1—C16—C17—O3	61.83 (18)
